# Novel porphyrazine-based photodynamic anti-cancer therapy induces immunogenic cell death

**DOI:** 10.1038/s41598-021-86354-4

**Published:** 2021-03-30

**Authors:** Victoria D. Turubanova, Tatiana A. Mishchenko, Irina V. Balalaeva, Iuliia Efimova, Nina N. Peskova, Larisa G. Klapshina, Svetlana A. Lermontova, Claus Bachert, Olga Krysko, Maria V. Vedunova, Dmitri V. Krysko

**Affiliations:** 1 Institute of Biology and Biomedicine, National Research Lobachevsky State University of Nizhny Novgorod, Nizhny Novgorod, Russian Federation; 2 Cell Death Investigation and Therapy Laboratory, Department of Human Structure and Repair, Ghent University, C. Heymanslaan 10, Building B3, 4th Floor, 9000 Ghent, Belgium; 3 Cancer Research Institute Ghent, Ghent, Belgium; 4 G.A. Razuvaev Institute of Organometallic Chemistry of the Russian Academy of Sciences, Nizhny Novgorod, Russian Federation; 5 Upper Airways Research Laboratory, Department of Head and Skin, Ghent University, Ghent, Belgium

**Keywords:** Cancer immunotherapy, Cell therapies, Biophotonics, Cell death and immune response, Immunology, Immune cell death

## Abstract

The immunogenicity of dying cancer cells determines the efficacy of anti-cancer therapy. Photodynamic therapy (PDT) can induce immunogenic cell death (ICD), which is characterized by the emission of damage-associated molecular patterns (DAMPs) from dying cells. This emission can trigger effective anti-tumor immunity. Only a few photosensitizers are known to induce ICD and, therefore, there is a need for development of new photosensitizers that can induce ICD. The purpose of this work was to analyze whether photosensitizers developed in-house from porphyrazines (pz I and pz III) can induce ICD in vitro and in vivo when used in PDT. We indetified the optimal concentrations of the photosensitizers and found that, at a light dose of 20 J/cm^2^ (λ_ex_ 615–635 nm), both pz I and pz III efficiently induced cell death in cancer cells. We demonstrate that pz I localized predominantly in the Golgi apparatus and lysosomes while pz III in the endoplasmic reticulum and lysosomes. The cell death induced by pz I-PDT was inhibited by zVAD-fmk (apoptosis inhibitor) but not by ferrostatin-1 and DFO (ferroptosis inhibitors) or by necrostatin-1 s (necroptosis inhibitor). By contrast, the cell death induced by pz III-PDT was inhibited by z-VAD-fmk and by the necroptosis inhibitor, necrostatin-1 s. Cancer cells induced by pz I-PDT or pz III-PDT released HMGB1 and ATP and were engulfed by bone marrow-derived dendritic cells, which then matured and became activated in vitro. We demonstrate that cancer cells, after induction of cell death by pz I-PDT or pz III-PDT, are protective when used in the mouse model of prophylactic tumor vaccination. By vaccinating immunodeficient mice, we prove the role of the adaptive immune system in protecting against tumours. All together, we have shown that two novel porphyrazines developed in-house are potent ICD inducers that could be effectively applied in PDT of cancer.

## Introduction

The emergence of immunotherapy as an independent and effective anti-neoplastic strategy has transformed the treatment of cancer with prospects for long-term tumor control, even in individuals with advanced-stage disease^1,2^. The immunogenicity of dying cancer cells has recently been recognized as a critical determinant of the efficacy of cancer therapy. In this context, a new useful paradigm for anti-cancer therapy is immunogenic cell death (ICD), which triggers activation of an immune response leading to a strong and long-lasting anti-cancer immunity specific for the host cancer cells^3,4^. ICD is characterized by the emission of danger-signaling molecules named damage-associated molecular patterns (DAMPs). These molecules are normally hidden within the cells, and in normal conditions they are involved in different physiological processes^5^. But during cell death, they are either exposed on the surface of dying cells (e.g., calreticulin) or liberated from the dying cancer cells (e.g., HMGB1, ATP). Once released, they function as adjuvants that activate antigen-presenting cells (e.g., dendritic cells), which engulf dying cancer cells and cross-present antigenic peptides on major histocompatibility complex class I (MHC I) molecules to CD8^+^ T cells. ICD can be induced in cancer cells by chemo-, radio-, or photodynamic therapy^3,4,6,7^.

Photodynamic therapy (PDT) is a minimally invasive, clinically approved therapeutic modality that has been added to the list of strategies that can induce ICD in various cells^8,9^. PDT has been known from the early days of the twentieth century when dyes such as eosin were used together with light to treat skin cancer^10,11^. PDT is based on the administration of a photodynamic agent (photosensitizer) and the action of intracellular oxygen, followed by exposure to light of a wavelength corresponding to that of the photosensitizer absorption band. The activated photosensitizer can then transfer the energy to molecular oxygen to produce singlet oxygen. The highly reactive toxic species rapidly react with cellular components, leading to a series of events, including direct tumor cell death, damage to the microvasculature, and induction of a local inflammatory reaction^12^. For PDT to be effective in anti-cancer therapy, it should fulfill several requirements: (1) the photosensitizer should accumulate selectively in tumors; (2) it should have low dark toxicity and be easy to synthesize; (3) it should be capable of inducing ICD.

**Figure 1:**
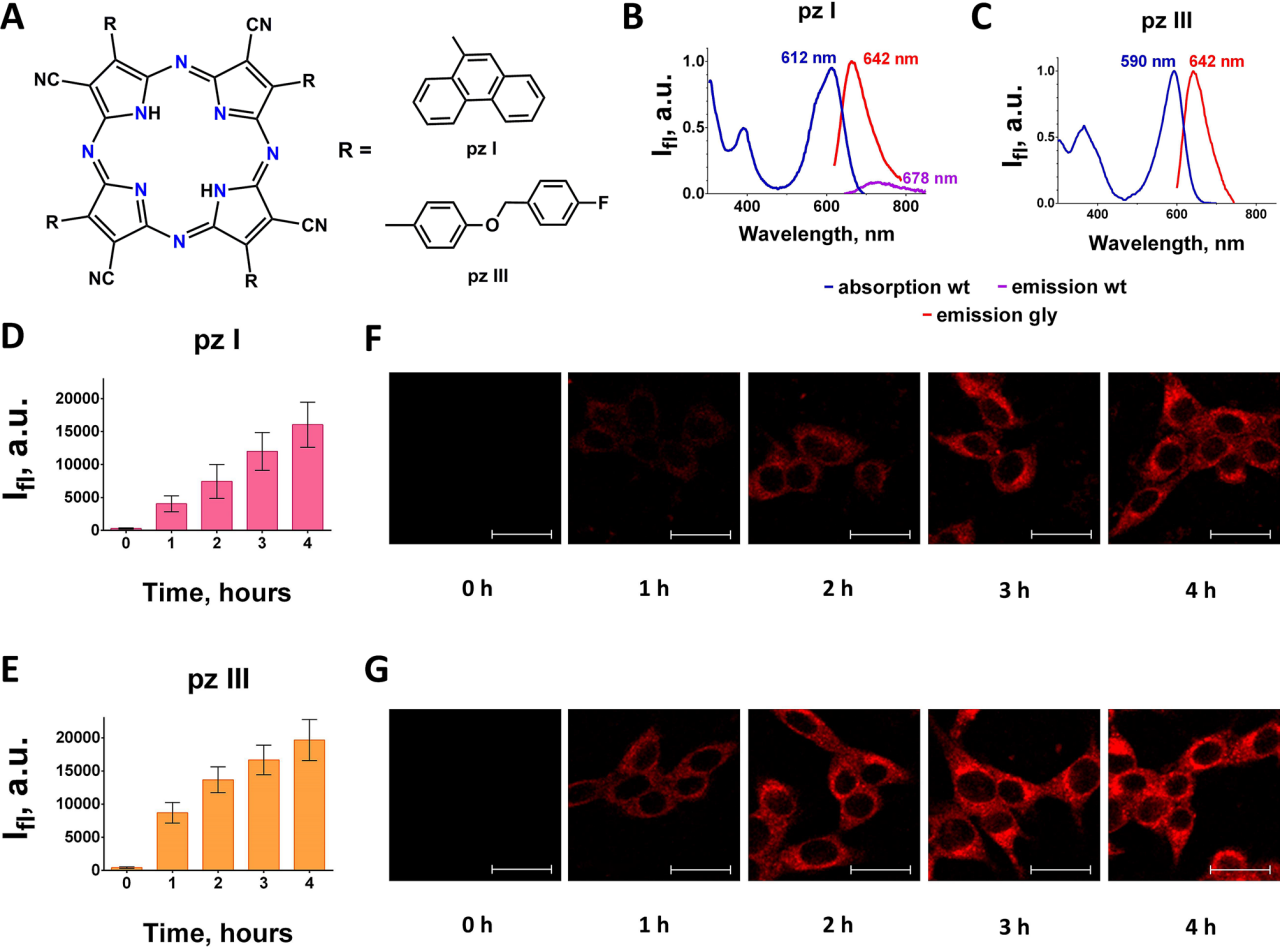
(**A**) Chemical structure of cyanoarylporphyrazines. (**B**, **C**) Fluorescence spectra and absorbance were obtained by spectrofluorometry for pz I (**B**) and pz III (**C**). Since pz I and pz III have a very low quantum yield of fluorescence in low-viscosity media (water), the pz I and pz III fluorescence was analyzed in a glycerol solution (in various ethanol:glycerol ratios). (**D, E**) Quantification of the fluorescence signal in fibrosarcoma MCA205 cells incubated with pz I or pz III expressed as mean ± SD (n = 5). The background intensity (Ifl) of the fluorescence signal before the addition of the photosensitizers did not exceed 0.3 a.u. (**F**, **G**) The uptake of pz I and pz III by fibrosarcoma MCA205 cells was quantified by confocal microscopy. The uptake was analyzed during up to 4 h of incubation with 10 μM of each photosensitizer. Images were acquired at λex 561 nm and λ_em_ 600–700 nm; scale bars 20 μm.

Recent years have witnessed a revival of PDT, with efforts focused on developing novel photosensitizers that that can mediate PDT of cancer-based on ICD induction^9,13–18^. Recently, we have also shown that clinically approved photosensitizers (*i.e*., photosens and photoditazine) could be effectively used to trigger ICD and combined with PDT in cancer therapy^17^. But there is still a need for the development of new photosensitizers that can induce ICD. Recent research has shown the advantages of newly synthesized fluorescent dyes derived from the porphyrazines group^19,20^. The unique feature of tetracyanoporphyrazines (porphyrazines, pz), which are tetrapyrrole macrocycles similar to porphyrins and phthalocyanines, is the combination of high photodynamic activity with high sensitivity to the viscosity of the medium^21^, along with excellent uptake and retention properties^22,23^. Moreover, in our previous study^20^, we determined that porphyrazines effectively induce cell death in cancer cells but are not toxic to normal, non-transformed cells. Therefore, the aim of the present study was to investigate whether the two cyanoarylporphyrazines (pz I and pz III) we developed in-house (Fig. 1A) can induce ICD in vitro and in vivo in a tumor prophylactic vaccination model.

In this study, we analyzed the cell death type induced by pz I-PDT and pz III-PDT and studied their intracellular accumulation dynamics and subcellular distribution. Pz I localized in the Golgi apparatus and lysosomes while pz III in the endoplasmic reticulum (ER) and lysosomes. Importantly, we demonstrated that cancer cells liberate DAMPs such as ATP and HMGB1 and are phagocytosed by bone marrow-derived dendritic cells (BMDCs) in a ratio-dependent manner. Dying cells cause activation of BMDCs in vitro. Finally, cancer cells stimulated with pz I-PDT or pz III-PDT protected mice in a tumor prophylactic vaccination model which was dependent on the activation of the adaptive immune system.

## Results

### Pz I and pz III: spectral characteristics, accumulation dynamics and subcellular distribution in cancer cells

In aqueous solution, all the absorption peaks of pz I and pz III were in both the shortwave region (Soret band) and the long‐wave region (Q‐band) of the spectrum, which is in agreement with previous reports (Fig. 1B,C)^20^. Pz III contains an additional peripheral aryl substituent that contributes to the total π‐conjugated aromatic system of the macrocycle and leads to the bathochromic shift of Q‐band (Fig. 1C). In a high viscosity environment, the fluorescence intensities of pz I and pz III increased greatly because viscous media prevent segmental intermolecular mobility. Therefore, their fluorescence was analyzed in a glycerol mixture. The absorbance spectra were analyzed in different media, including glycerol and water (Table 1). Pz I and pz III accumulated in MCA205 cells during in vitro incubation. Their uptake rates were slightly different (Fig. 1D–G). Of note that incubation for 4 h was enough for both photosensitizers to accumulate a significant extent in MCA205 cells and this incubation time was chosen for analysis of their photodynamic activities.

**Table 1:** Spectral characteristics of the photosensitizers.

Photosensitizer	λ_abs_, nm*	ε (l × mol^−1^ × cm^−1^)	λ_em_ (nm*)
pz I	612 wt	29 × 10^−4^	678 wt
			642 gly
pz III	590 gly	2.1 × 10^−4^	642 gly

*As pz I and pz III showed very weak fluorescence in water (wt), their fluorescence was analyzed in an ethanol–glycerol (gly) mixture with a viscosity of about 45 cP.

**Figure 2:**
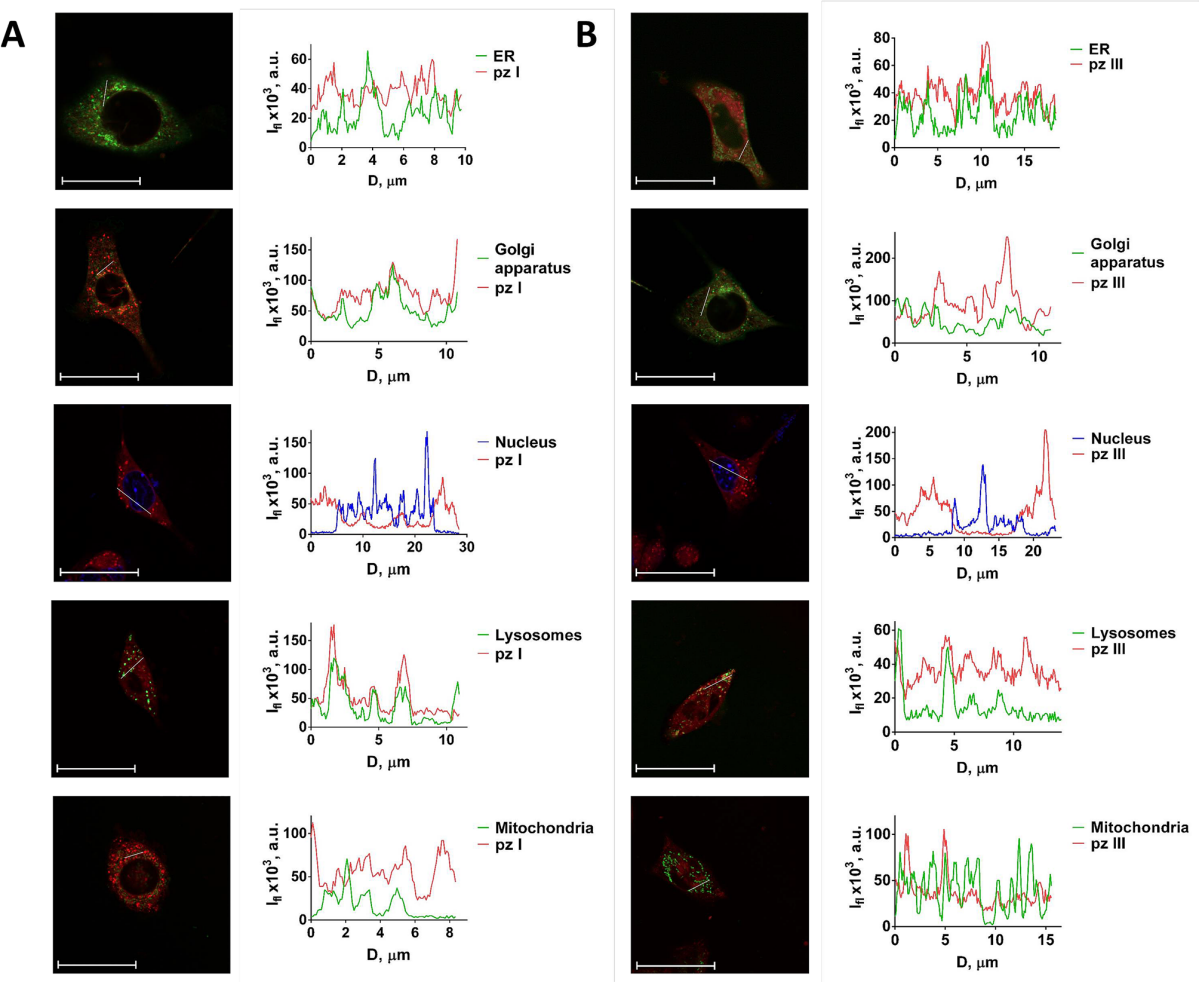
Analysis of subcellular distribution of pz I and pz III by confocal microscopy. The subcellular localization of pz I (**A**) and pz III (**B**) was studied by confocal microscopic examination of fibrosarcoma MCA205 cells after 4 h of incubation with 10 μM of the photosensitizers. Pz I localizes primarily in the Golgi apparatus and partially in the lysosomes (**A**), while pz III accumulates mainly in the ER, and there is a connection with lysosomes (**B**). Fluorescence signal profiles along the lines are indicated by the white arrow on the images with superimposed fluorescence channels. D: distance along the specified segment. Ifl: fluorescence intensity. The following dyes were used: LysoTracker Green (lysosomes), MitoTracker Green (mitochondria), ER-Tracker (ER), BODIPY FL C5-ceramide (Golgi apparatus), and DAPI (nucleus).

The ability of PDT to induce ICD often depends on the localization of the photosensitizer to the ER^9,24–27^. Therefore, the intracellular spatial distribution of pz I and pz III in fibrosarcoma MCA205 cells was detected by laser scanning confocal microscopy (Fig. 2). In the images, the co-localization of pz I and pz III with dyes that specifically localize in specific organelles was examined by comparing the signal distribution profiles in the corresponding fluorescent channels. Of note, that pz I localized primarily in the Golgi apparatus and partially in the lysosomes (Fig. 2A). Pz III accumulated mainly in the ER, and there is a connection with lysosomes (Fig. 2B). Importantly, neither photosensitizer was detected in mitochondria and the nucleus. Therefore, several mechanisms might be involved in ICD besides those associated with ER^3,25,27,28^. Notably, ER independent mechanisms of immunogenicity have also been described^29^.

### PDT based on pz I and pz III efficiently induces cell death in cancer cells

**Figure 3:**
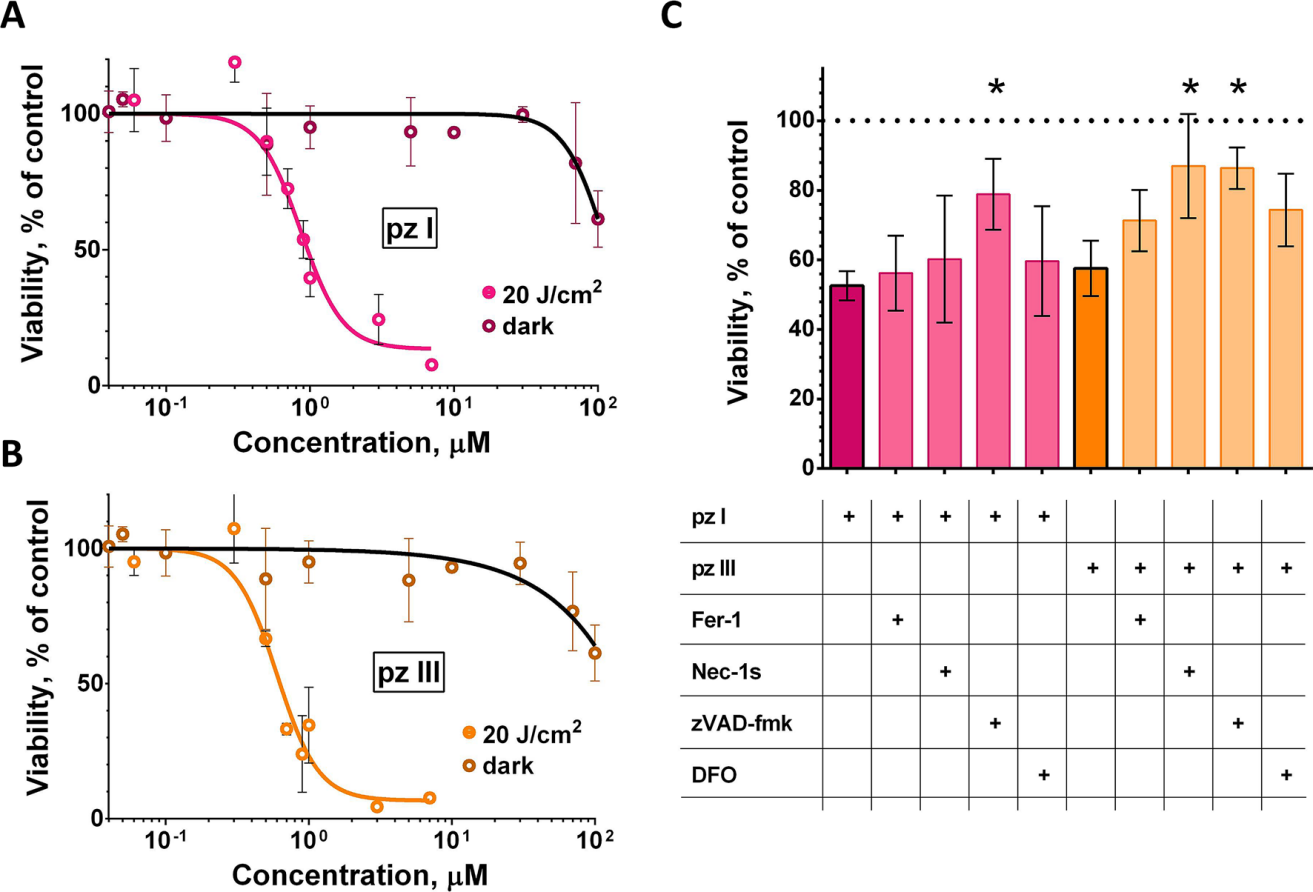
Quantification of cell death after treatment with pz I-PDT or pz III-PDT by MTT assay. (**A**, **B**) Dark toxicity (black lines) was assessed in fibrosarcoma MCA205 cells after addition of the photosensitizer in serum-free medium for 24 h. Cell death was induced by incubating the cells with 0.05–7.0 µM photosensitizer in serum-free medium for 4 h and then exposing them to a light dose of 20 J/cm^2^ with power density of 20 mW/cm^2^ from a LED light source (615–635 nm). The MTT assays were performed 24 h after irradiation. ^#^IC_50_ for pz I was 2.0 μM [1.85–2.11] and for pz III 1.1 μM [0.97–1.2]. The values were calculated with 95% confidence intervals (three individual experiments with three replicates in each). (**C**) Effect of different inhibitors on the death of MCA205 cells induced by pz I-PDT or pz III-PDT. The following inhibitors were used to block cell death induced by pz I-PDT or pz III-PDT: 25 μM zVAD-fmk (apoptosis), 20 μM necrostatin-1 s (necroptosis), and 1 μM ferrostain-1 or 10 μM DFO (ferroptosis). Cell death in fibrosarcoma MCA205 cells induced by pz I-PDT was significantly blocked by zVAD-fmk. In contrast, cell death induced by pz III-PDT was inhibited by either zVAD-fmk or necrostatin-1 s. In this procedure, the cells were pre-incubated with 2.0 μM of pz I or 1.1 μM of pz III in the presence of the respective cell death blocker in serum-free medium for 4 h. The medium was then replaced by photosensitizer-free medium followed by irradiation at 20 J/cm^2^ using a LED light source (615–635 nm). The respective inhibitor was added again after irradiation. Thirteen hours after irradiation MTT assays were performed. Dotted line represents untreated control (no photosensitizer or inhibitor; set as 100% viable cells). The values are the means ± SEM from n = 3. t-criteria with Bonferroni correction were used to calculate statistical significance, *p < 0.05; ^#^IC_50_ values are the mean values ± SEM.

The dark toxicity of pz I and pz III was tested for 24 h without irradiation on fibrosarcoma MCA205 cells (Fig. 3A,B). The colorimetric assay based on 3-(4,5-dimethylthiazol-2-yl)-2,5-diphenyltetrazolium bromide (MTT) showed that cell survival was > 90% for both photosensitizers. Cell death was not induced by pz I or pz III in concentrations of up to 7 μM in the dark (Fig. 3A,B). In concentrations above 20 μM, both photosensitizers induced significant dark toxicity. Based on the MTT assay, irradiation with a light dose of 20 J/cm^2^ induced cell death in fibrosarcoma MCA205 cells in a concentration-dependent manner. Cell death was induced by pz I and pz III at concentrations of 0.05 − 7.0 μM (Fig. 3A,B). After irradiation of MCA205 cells with a light dose of 20 J/cm^2^, pz I and pz III had IC_50_ values of 2.0 μM and 1.1 μM, respectively.

Next, to determine the cell death type, inhibitors of different cell death modalities were used: zVAD-fmk for apoptosis, Nec-1 for necroptosis, and Fer-1 and DFO for ferroptosis^17,30^. Previously, it has been shown that the application of these cell death inhibitors allows to specifically discriminate between different cell death modalities apoptosis, necroptosis and ferroptosis^30–32^. When MCA205 cells were killed by pz I-PDT, the addition of the zVAD-fmk inhibitor significantly reduced cell death, indicating that the induced cell death followed the apoptotic pathway (Fig. 3C). Importantly, the other blockers did not have an inhibitory effect on cell death after pz I-PDT treatment. However, in contrast to MCA205 cells killed by pz III-PDT, two inhibitors (zVAD-fmk and Nec-1 s) significantly reduced cell death. In this situation, all ferroptosis inhibitors (i.e*.*, Fer-1, DFO) were ineffective. These data indicate that pz I-PDT induces apoptosis while pz III-PDT triggers mixed types of cell death with features of apoptosis and necroptosis. Recently, it has been shown that mixed types of cell death can be induced by PDT^17,33^.

### Pz I-PDT and pz III-PDT induce immunogenic cell death in cancer cells: release of ATP and HMGB1

**Figure 4:**
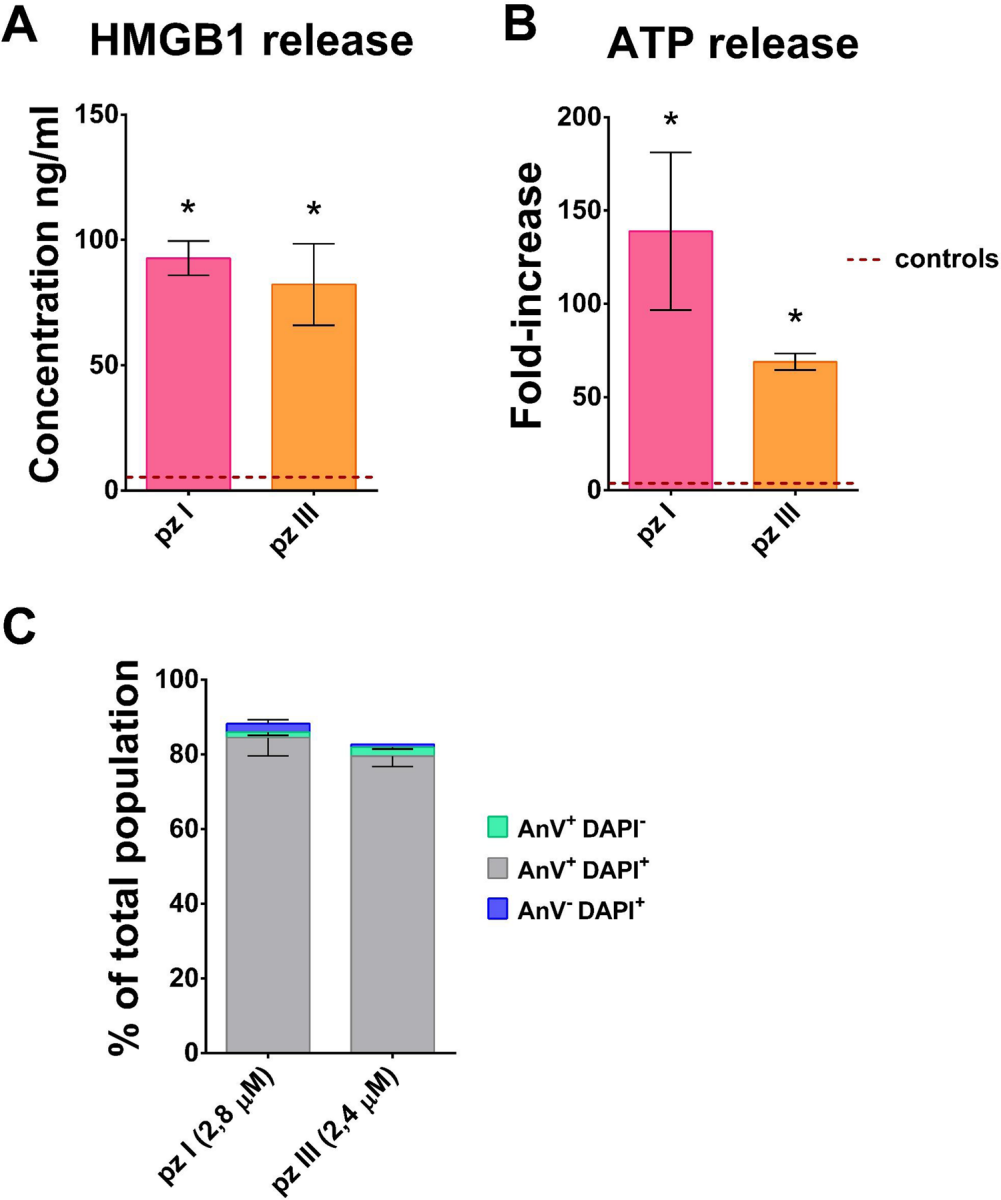
Cell death induced in cancer cells by pz I-PDT or pz III-PDT is associated with HMGB1 and ATP release. (**A**) HMGB1 release. Fibrosarcoma MCA205 cells were recovered for 24 h after treatment with 2.8 µM of pz I-PDT or 2.4 µM of pz III-PDT (IC_90_) or without treatment (live). HMGB1 was measured in the supernatants. HMGB1 values represent the mean values of five independent experiments. Statistical significance was calculated by Mann–Whitney nonparametric test, *p < 0.001. (**B**) ATP release. Fibrosarcoma MCA205 cells were recovered for 24 h after treatment with pz I-PDT or pz III-PDT or without treatment (live). In the supernatants ATP was measured and its values represent fold increase relative to untreated cells (mean values of six independent experiments). Statistical significance was calculated by using Mann–Whitney non-parametric test, *p < 0.006. (**C**) Cell death was measured by staining the MCA205 cells with Annexin-V/DAPI followed by flow cytometry. Cell death analysis was performed after 24 h of incubation after PDT irradiation. The representative flow cytometry dot plots of Annexin-V/DAPI stained MCA205 cells are shown in the Supplementary Fig. 1.

ICD is characterized by the coordinated emission of various DAMPs, such as ATP and HMGB1^34^. DAMPs function as adjuvants that contribute to the activation of antigen-presenting cells (e.g., dendritic cells), engulfing dying cancer cells and cross-presenting antigenic peptides to CD8^+^ T cells, one of the main driving forces of anti-tumor immune responses^35,36^. To determine the immunogenic nature of the cell death process induced by pz I-PDT or pz III-PDT, we examined the generation of several well-known DAMPs. We measured the release of ATP and HMGB1 into the extracellular environment after induction of cell death in fibrosarcoma MCA205 cells by pz I-PDT or pz III-PDT. The release of HMGB1 into the culture supernatant by dying MCA205 cells was confirmed by a sensitive ELISA (Fig. 4A). By 24 h after PDT, HMGB1 levels increased about 11-fold relative to untreated MCA205 cells. Using another assay, we found that stimulation of MCA205 cells with pz I-PDT or pz III-PDT triggered the release of ATP into the extracellular environment in amounts that were about 90-fold higher than seen in untreated MCA205 cells (Fig. 4B). Importantly, the emission of ATP and HMGB1 was concomitant with plasma membrane rupture (Fig. 4C, Supplementary Fig. 1), which points to passive DAMPs release. In conclusion, pz I-PDT and pz III-PDT can stimulate the release of two crucial DAMPs from cancer cells, demonstrating the immunogenic nature of the induced cell death.

### Cancer cells killed by pz I-PDT or pz III-PDT are phagocytosed and induce activation and maturation of BMDCs

Next, we analyzed by flow cytometry the in vitro phagocytosis of fibrosarcoma MCA205 cells killed by pz I-PDT or pz III-PDT. By co-culturing untreated MCA205 cells (viable cells) or cells treated with pz I-PDT or pz III-PDT MCA205 with primary BMDCs, we observed that dying cells were engulfed in a dose dependent manner by BMDCs, but viable cells were not (Supplementary Fig. 2). Together, these in vitro results indicate that pz I-PDT and pz III-PDT are potent inducers of the typical characteristics of ICD in fibrosarcoma MCA205 cells: the release of ATP and HMGB1 and clearance of the dead/dying cancer cells by BMDCs.

**Figure 5:**
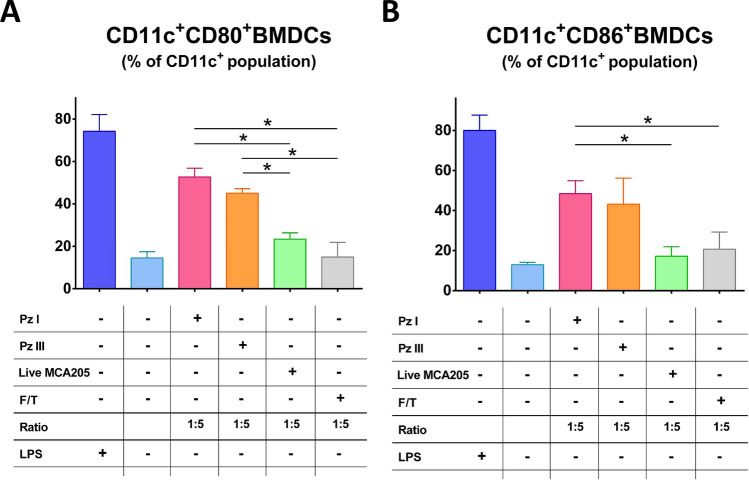
Cancer cells killed by pzI-PDT or pz III-PDT induce activation and maturation of BMDCs in vitro. BMDCs were cocultured with dying MCA205 cells treated either with pz I-PDT or pz III-PDT, or were cocultured with viable MCA205 cells at 1:5 ratio. Percentages of CD11c^+^CD80^+^ (**A**) and CD11c^+^CD86^+^ (**B**) BMDCs cells shown as mean values ± SEM of three independent experiments. BMDCs stimulated with LPS served as positive control while fibrosarcoma MCA205 cells subjected to the several cycles of freeze–thaw (F/T) were used as a negative control (at 1:5 ratio). Besides, BMDCs left untreated were used as a negative control. Statistical significance was calculated by a multiple t-test using Sidak-Bonferroni method, *p < 0.05. The representative dot plots, which show the gating strategy and percentage of CD11c^+^CD80^+^ and CD11c^+^CD86^+^ BMDCs in co-culture with MCA205 cells are shown in the Supplementary Figs. 3 and 4, respectively.

To analyze the functional status of BMDCs, we evaluated the immunogenic properties of MCA205 cells killed by pz I-PDT or pz III-PDT in vitro (Fig. 5). For this, we compared BMDCs co-cultured with cancer cells treated with pz I-PDT or pz III-PDT to BMDCs that were exposed to live cells. LPS was used as a positive control. Dying fibrosarcoma MCA205 cells treated with pz I-PDT induced phenotypic maturation of BMDCs, as indicated by surface upregulation of the co-stimulatory molecules CD80 (Fig. 5A, Supplementary Fig. 3) and CD86 (Fig. 5B and Supplementary Fig. 4) when compared with BMDCs co-cultured with live cells. Co-culture with the same amount of pz I-PDT or pz III-PDT treated dying MCA205 cells induced CD80 surface expression significantly higher compared with negative control BMDCs co-cultured with cells undergoing accidental necrosis (F/T cells, Fig. 5A). Remarkably, co-culture with the same amount of MCA205 cells subjected to F/T did not induce the maturation status of the BMDCs (Fig. 5). These data are in accordance with the previously published findings indicating that cancer cells killed by freeze–thaw cycles to induce accidental necrosis are only weakly immunogenic or not at all^17,31,35^. Accidental necrosis is a non-regulated cell death type that is normally triggered by harsh, external, physical or chemical stimuli, including heat, freeze-thawing cycles, mechanical stress, or osmotic shock. The term accidental necrosis is a well-accepted in the literature^31,35,37^.

### Vaccination with cancer cells killed by pz I-PDT or pz III-PDT induces effective adaptive anti-tumor immunity

**Figure 6:**
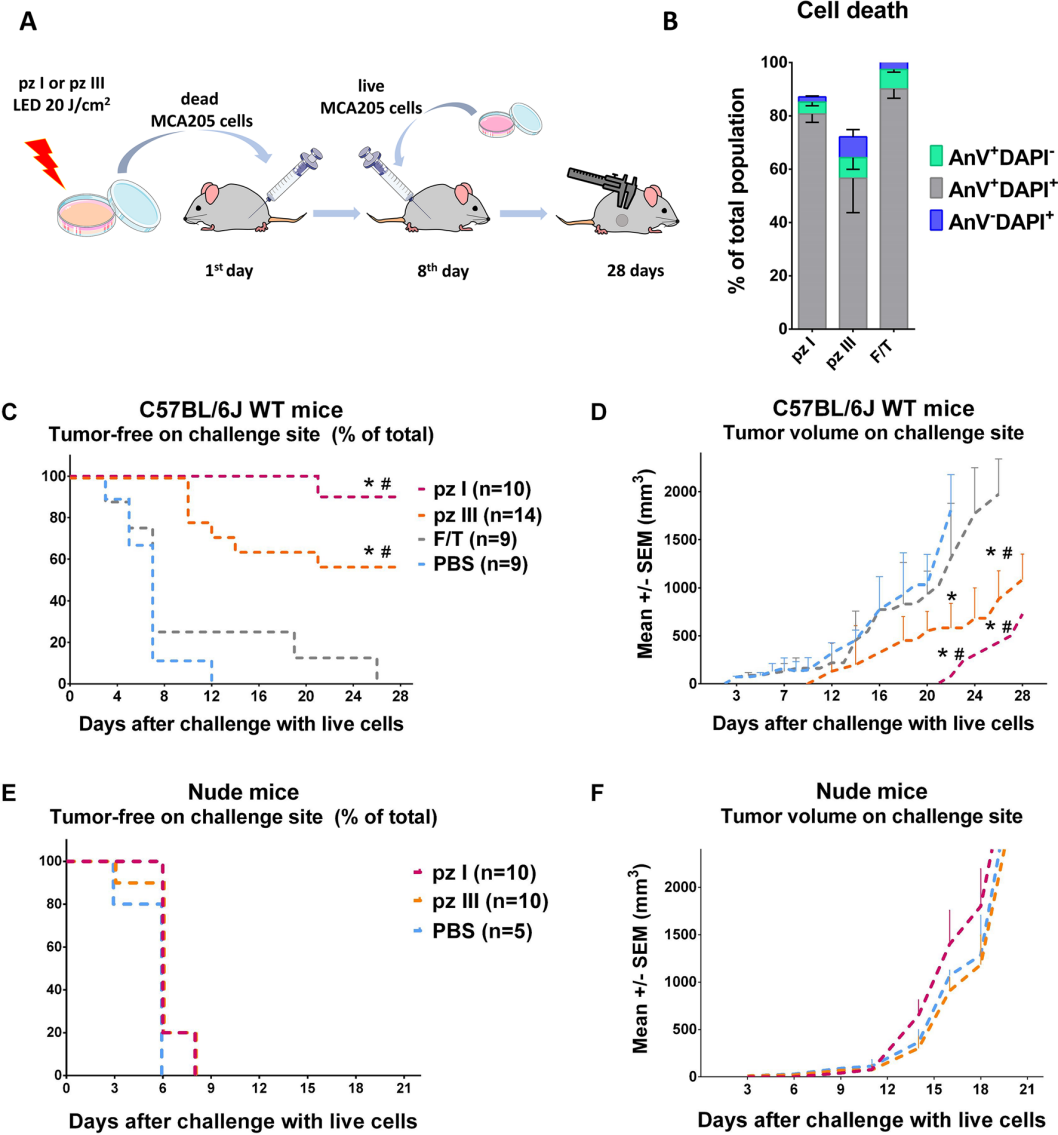
Cancer cells treated with pz I-PDT or pz III-PDT are immunogenic in vivo. (**A**) Scheme of in vivo prophylactic tumor vaccination model. Fibrosarcoma MCA205 cells were stimulated with IC_90_ concentration of pz I-PDT or pz III-PDT and re-suspended in PBS; 5 × 10^5^ cells were injected in mice. As a control, mice were injected either with PBS or with 5 × 10^5^ MCA205 cells subjected to several free-thaw cycles (F/T). One week later, the mice were challenged with 1 × 10^5^ viable MCA205 cells in the other flank. The percentage of mice with tumor‐free challenge flank was determined (**C**, **E**) and tumor growth at the challenge flank was measured (**D**, **F**). (**B**) Cell death was measured by staining the MCA205 cells used for immunization with Annexin-V/DAPI followed by flow cytometry. The representative flow cytometry dot plots of Annexin-V/DAPI stained MCA205 cells are shown in the Supplementary Fig. 5. (**C**) The evolution of tumor incidence over time in C57BL/6 J mice (wild type, WT) is shown as a Kaplan–Meier curve. Dying fibrosarcoma MCA205 cells induced by pz I-PDT or pz III-PDT triggered an efficient anti-tumor immune response. Of note, tumors grew rapidly at the challenge site on mice vaccinated with F/T cells or PBS. The statistical difference from PBS (*) or F/T (#) immunization (negative controls) was calculated by a long-rank Mantel-Cox test, *^,#^*p* < 0.01. (**D**) In the prophylactic tumor vaccination experiments the size of the tumors growing at the challenge site of the C57BL/6 J wild type (WT) mice. (**E**, **F**) The immunization of Nude mice was done as described in (**A**). No antitumor immune response was triggered in Nude mice immunized with MCA205 cells treated with pz I-PDT or pz III-PDT. The statistical differences from PBS group or mice vaccinated with cells subjected to F/T are shown for each vaccination group and were calculated by a Mann–Whitney nonparametric t-test, *^,#^*p* < 0.05. The difference from *PBS and from ^#^F/T groups.

To investigate the ability of cancer cells killed by pz I-PDT or pz III-PDT to activate the adaptive immune system, we carried out in vivo experiments in immunocompetent C57BL/6 J mice^4,17,38–40^. We immunized the mice with dying/dead fibrosarcoma MCA205 cells treated with pz I-PDT or pz III-PDT. As a negative control, we injected mice with PBS or with MCA205 cells undergoing accidental necrosis^17,41,42^. The immunized C57BL/6 J mice were then challenged with live fibrosarcoma MCA205 cells. Protection at the challenge site against tumor growth was interpreted as a sign of successful priming of the adaptive immune system (Fig. 6A,B and Supplementary Fig. 5). C57BL/6 J mice immunized with MCA205 cells treated with pz I-PDT or pz III-PDT showed strong signs of activation of the adaptive immune system: both treatments strongly prevented the tumor growth seen in the non‐immunized mice. In contrast, most of the control C57BL/6 J mice immunized with MCA205 cells subjected to two F/T cycles showed tumor growth after a challenge similar to that seen in mice immunized with PBS (Fig. 6C,D). That observation confirms the poor immunogenic properties of cancer cell death by accidental necrosis^17,35,43^. In addition, the tumors growing at the challenge site of the mice vaccinated with cancer cells stimulated with pz I-PDT or pz III-PDT were significantly smaller than the tumors on the mice immunized with PBS or with cells undergoing accidental necrosis. In order to evaluate the role of the adaptive immune system in immunogenicity of MCA205 cells treated with pz I-PDT or pz III-PDT, Nude mice were vaccinated (Fig. 6E,F). Severe T cell deficiency prevents mice from developing adaptive immunity in response to vaccination with dying MCA205 cells. Overall, these data confirm that ICD is induced in cancer cells by treatment with pz I-PDT or pz III-PDT and that such treated cells induce a potent adaptive immune response in vivo.

## Discussion

We show that pz I-PDT and pz III-PDT induce ICD in cancer cells. We determined the IC_50_ for both photosensitizers and also found that pz I was localized predominantly in the Golgi apparatus and lysosomes while pz III in the ER and lysosomes. Next, we showed that the cell death induced in cancer cells by pz I-PDT or pz III-PDT is associated with the release of the two crucial DAMPs, ATP and HMGB1, and that these dying cancer cells are efficiently engulfed in vitro by BMDCs and induce their activation and maturation. Finally, those in vitro findings were confirmed in vivo by showing that fibrosarcoma MCA205 cells stimulated with pz I-PDT or pz III-PDT served as a potent vaccine in a prophylactic tumor vaccination model. The role of adaptive immunity in vaccination was demonstrated in immune compromised Nude mice. These findings identify pz I-PDT and pz III-PDT as novel inducers of ICD and open new prospects for the use of these novel photosensitizers to efficiently kill cancer cells and use them as a vaccine to initiate immune responses leading to effective anti-tumor immunity.

PDT is a clinically approved, minimally invasive therapeutic procedure that can be selectively cytotoxic to malignant cells^9,12^. Several different photosensitizers or their precursors have been identified and shown to efficiently induce ICD^15–17,20,25,44^. In this study, we analyzed the ICD-inducing potential of in-house developed porphyrazines, a class of macrocycles that combine some of the features of porphyrins and phthalocyanines^22,45^. It is also important to emphasize that pz I-PDT and pz III-PDT were nontoxic in MCA205 cancer cells in the absence of light irradiation, a property photosensitizer must possess in order to avoid unwanted side effects when administered to patients^46^. Interestingly, though both pz I and pz III belong to the same class of porphyrazines (Fig. 1A), they induce different cell death modalities in cancer cells. We found that the apoptosis inhibitor zVAD-fmk significantly blocked the cell death induced by pz I-PDT. In contrast, the cell death induced by pz III-PDT was blocked by necrostatin-1 s and by zVAD-fmk, which indicates that pz III-PDT induces mixed features of apoptosis and necroptosis in cancer cells. Indeed, the immunogenicity of necroptosis has been described^35,47–50^. These data are in line with our previously published study in which we showed that PDT can induce mixed cell death phenotypes^17^. The notion that a single photosensitizer can trigger more than one cell death phenotype is very attractive because this strategy may be useful for overcoming the frequently observed resistance of cancer cells to a single cell death type^51–53^. To overcome cell death resistance, the use of two photosensitizers has been suggested in order to provide a synergistic, curative and highly effective strategy that triggers two cell death types^18,33^. Both pz I and pz III have proven to be efficient inducers of cell death not only in fibrosarcoma MCA205 cells, but also in other cancer cell lines, such as glioma GL261 cells^20^.

The ability to chemically induce ICD is often associated with the localization of the drugs or photosensitizers in the ER and their ability to induce ER stress. It has been reported that photosensitizers can be localized in the ER and thereby cause strong ICD induction after PDT^3,24–26^. In this study, we found that pz III was predominantly accumulated in the ER and lysosomes, confirming the previously published studies on the close relationship between ER stress and ICD. Importantly, if photosensitizers do not accumulate in the ER (e.g*.*, indocyanine green, which is found mainly in the cytoplasm after cellular internalization^54^), the efficiency of ICD induction might be reduced. Several ER-targeting strategies have been proposed to overcome this limitation^26,27^.

The results presented here add two novel in-house developed photosensitizers from the group of porphyrazines, pz I and pz III, to the list of compounds that can efficiently induce ICD. This broadens the potential application of ICD in cancer treatment. Pz I localizes primarily in the Golgi apparatus and partially in the lysosomes, while pz III accumulates mainly in the ER, and there is a connection with lysosomes. These photosensitizers induce typical features of ICD, such as emission of two crucial DAMPs, ATP and HMGB1. The role of calreticulin, which is another crucial DAMP, was not addressed in this study, and it is conceivable that calreticulin might contribute to the immunogenic potential of dying cancer cells killed by pz I-PDT or pz III-PDT^42^. The cancer cells killed by pz I-PDT or pz III-PDT are effectively engulfed, leading to phenotypic maturation and activation of BMDCs. In vivo, cancer cells killed by pz I-PDT or pz III-PDT protect mice from tumor growth. Moreover, in vivo experiments in Nude mice have shown that tumors in the pz I and pz III groups appeared and developed as quickly as in the negative control group injected with PBS. These findings indicate that the adaptive immune system is essential to induce tumor protection in this mouse model which is in line with the previously published findings on the role of the adaptive immune system in vaccination with apoptotic^41^, necroptotic^48–50^ and ferroptotic^31^ tumor cells. Thus, our findings point to PDT as an effective method to induce ICD, and future studies will shed light on the role of the ICD induced by PDT in humans. That may lead to trials to fully explore the immunological potential of PDT as an effective treatment of many cancer patients.

## Materials and methods

### Cell lines

Murine fibrosarcoma MCA205 cells were obtained from VIB-UGent Center for Inflammation Research (Ghent, Belgium). They were grown in RPMI medium (GIBCO) supplemented with 4.5 g/L glucose, 2 mM glutamine (GIBCO), 100 μM sodium pyruvate, 100 units/ml penicillin, 100 μg/L streptomycin and 10% fetal bovine serum (FBS, Fisher Scientific).

### Spectra acquisition

The following in-house made photosensitizers were used: (1) pz I (cyanoarylporphyrazines with 9-phenanthrenyl group in the aryl frame of the macrocycle) and (2) pz III [4-(4-fluorobenzyoxy)phenyl group in the aryl frame] (patent RU2672806C1, Russia) (Fig. 1A). Black 96-well microplates with a clear glass bottom (Falcon Imaging; Corning) were used to analyze the pz I and pz III absorbance spectra and fluorescence emission by using a Synergy MX Microplate Reader (BioTek). Distilled water was used to prepare 9.09 µM (10 μg/ml) pz I and 8.33 µM (10 μg/ml) pz III solutions. Fluorescence was excited at 590 nm. Since the fluorescence quantum yield of pz I and pz III in non-viscous media is very low, the fluorescence was measured in an ethanol-glycerol solution (2:3).

### Induction of cell death by pz I- or pz III-based PDT

PDT based on pz I or pz III was applied to cancer cells to induce cell death. Fibrosarcoma MCA205 cells were first incubated in a serum-free culture medium for 4 h with 2.0 μM pz I or 1.1 μM pz III (IC_50_). The cells were irradiated for 16 min 40 s to achieve a dose of 20 J/cm^2^ using a LED light source (λex 630 nm) in a serum-free culture medium without photosensitizers. Importantly, the cells were handled either in the dark or in subdued light after the addition of the photosensitizers. Following irradiation, the cancer cells were cultured in full culture medium supplemented with 10% FBS for the indicated duration period of time and cell death was analyzed by either MTT assay or flow cytometry. For control, cells were incubated in the same conditions but without photosensitizers and were not subjected to PDT.

Cell death was blocked by using the following pharmacological agents: the pan-caspase inhibitor carbobenzoxy-valyl-alanyl-aspartyl-[O-methyl]-fluoromethylketone (zVAD-fmk, 25 μM, Sigma-Aldrich), RIPK1 inhibitor necrostatin-1 s (Nec-1 s, 20 μM, Abcam), the inhibitor of ROS and lipid peroxidation ferrostatin-1 (Fer-1, 1 μM, Sigma-Aldrich) and the iron chelator, deferoxamine (DFO, 10 μM, Sigma-Aldrich). The corresponding photosensitizer and the cell death inhibitors were added simultaneously for 4 h and cells were incubated in serum-free conditions as described above. Just before PDT, the medium was replaced by a complete culture medium containing the respective cell death inhibitor, and the cells were irradiated with light at 20 J/cm^2^. After incubation of the cells for 24 h, MTT assays or flow cytometry were performed.

### Cell death assay by flow cytometry

For flow cytometry, the cells were first removed from the flask with 0.05% trypsin solution, centrifuged, washed in Annexin-V binding buffer, and stained with 1 µM DAPI (Thermo Fisher Scientific) and Annexin-V- FITC (Invitrogen). The assays were run on a BD FACSCanto flow cytometer. The data were analyzed using FlowJo software (v.10.0.8). The Annexin-V^−^/DAPI^−^ population reflects viable cells. The Annexin-V^+^/DAPI^−^ population is characterized by phosphatidylserine exposure at the cell surface with preserved integrity of the plasma membrane. The cells in this population are at the early cell death stage. The Annexin-V^+^/DAPI^+^ population reflects dying cells in the later stages. The Annexin-V^-^/DAPI^+^ population is a very minor population representing cells, which undergo cell death during the recording of the samples on a flow cytometer (Figs. 4, 6B, Supplementary Figs. 1 and 5). The Annexin-V/DAPI staining can detect only the stage of cell death but not the cell death type^37,55–58^.

### Cell death analysis by MTT

For the MTT assay (AlfaAesar), the cell culture medium was replaced with a serum-free medium with 10% MTT agent for 4 h. Then the medium was replaced with DMSO solution and the cells were shaken for 10 min. Optical density was measured at 570 nm in a Synergy MX Microplate Reader (BioTek).

### Accumulation dynamics and subcellular distribution of photosensitizers

The localization of photosensitizers in cytoplasmic organelles during PDT plays a major role in their ability to induce ICD. To examine this, murine fibrosarcoma MCA205 cells were treated with pz I or pz III and analyzed by using a LSM 710 Axio Observer Z1 DUO NLO laser scanning microscope (Carl Zeiss, Germany). Images were acquired with a LD C-Apochromat water immersion objective lens 40x/1.1. The fibrosarcoma MCA205 cells were seeded at a density of 10^4^ cells per well in 96-well glass-bottom plates (Corning) and were grown overnight. Next, the cells were incubated with 10 μM photosensitizers in a serum-free culture medium for 4 h, followed by washing with PBS. Fluorescence was excited at 594 nm and recorded at 600–670 nm. Confocal images were acquired.

To determine the subcellular localization of these photosensitizers after they were incubated for 3.5 h with fibrosarcoma MCA205 cells, the following dyes were added for 30 min (ThermoFisherScientific) according to the manufacturer's instructions: 0.5 μM LysoTracker Green DND-26 for lysosomes, 0.5 μM ERTracker for ER, 0.5 μM MitoTracker Green FM for mitochondria, 5 μM BODIPY FL C5-ceramide complexed with BSA for Golgi apparatus, and 3,0 μM DAPI for nucleus. Excitation was done by an argon laser at 488 nm and fluorescence was registered in the range of 500–560 nm.

### HMGB1 release

Murine fibrosarcoma MCA205 cells were treated with 2.8 µM pz I-PDT or 2.4 µM pz III-PDT (IC_90_) as described above. After the indicated durations, the supernatant was collected, cleared from dying tumor cells by centrifugation, and frozen at − 20 °C until HMGB1 was quantified later with an ELISA kit (IBL-Hamburg). All assays were performed in accordance with the manufacturer’s instructions. HMGB1 was quantified by using Tecan Spark 20 M microplate multimode reader. A four-parameter logistic curve fit was used for data analysis.

### ATP release

Murine fibrosarcoma MCA205 cells were incubated for 24 h with 2.8 µM pz I or 2.4 µM pz III (IC85) in RPMI medium supplemented with 2% FBS as described above. The supernatants were collected and centrifuged at 15,000 rpm at 4 °C for 3 min and then stored at -80 °C for ATP measurement if that was not done immediately. ATP analysis was done according to the manufacturer’s instructions by using CellTiter-Glo Luminescent Cell Viability Assay kit (Promega, G7571). Tecan Spark 20 M microplate multimode reader was used to quantify the luminescence.

### Isolation of mouse bone-marrow-derived dendritic cells

BMDCs were isolated from the femurs and tibias of C57BL/6 J mice at the age of 7–10 weeks. BMDCs were cultured for 10 days in RPMI medium (GIBCO) supplemented with 5% heat-inactivated fetal calf serum, 20 ng/ml mGM-CSF, 1% L-glutamine, 50 μM 2-mercapthoethanol, and 1 mM pyruvate. On days 3, 6 and 9, the culture medium was refreshed.

### Phagocytosis assay

To quantify the uptake of dead/dying cancer cells by BMDCs, we used a previously described two-parameter flow cytometry phagocytosis assay^17^. Fibrosarcoma MCA205 cells were stained with 1 μM CellTracker Green CMFDA (Molecular Probes) in a serum-free medium (30 min). After this, the target cancer cells were either left untreated or were induced to die by pz I-PDT or pz III-PDT, as described above. The cancer cells were then collected, washed, and co-cultured with BMDCs in ratios of 1:1 and 1:5 for 2 h. The co-cultures were harvested and immunostained with a mix of mouse Fc block (ThermoFisherScientific) and PE-Cy-anti-CD11c (BD PharMingen, 561,022). The BMDCs were finally analyzed by flow cytometry on a BD FACSCanto. Uptake was quantified with FlowJo software (v.10.0.8). A gating strategy that enables analysis of single cells was used in order to determine the actual uptake of CMFDA-labeled dead cell material by BMDCs. The percentage of CD11c CMFDA double-stained BMDCs in the total BMDC population represents the fraction of the BMDC population involved in the phagocytosis of target cells (% phagocytosis).

### Analysis of BMDC activation and maturation in vitro

Immature murine BMDCs were isolated and cultured as described previously^17,31^. Then BMDCs were co-incubated with dying fibrosarcoma MCA 205 cells stimulated with pz I-PDT or pz III-PDT as described above in ratios of 1:5 for 18 h. As a positive control, BMDCs were stimulated in parallel with 100 ng/ml of *E. coli* lipopolysaccharide (LPS^17,31^). After co-culture for 18 h, the cells were collected, spin down (400 × g, 6 min, 4 °C), and washed once in phosphate-buffered saline (PBS, Life Technologies) as described previously^17,31^. Dead cells were excluded from the flow cytometry analysis by staining with DAPI (Thermo Fisher Scientific) and Annexin-V- FITC (Invitrogen) as described above. Maturation of BMDCs was analyzed by immunostaining with PE-Cy-anti-CD11c (BD PharMingen), APC- or eFluor 450-anti-CD86 (eBioscience), eFluor 45-anti-CD80 (Thermo Fisher Scientific) and mouse Fc block (Thermo Fisher Scientific) as described previously^17,31^.

### In vivo prophylactic tumor vaccination

All in vivo experiments were performed in accordance with the guidelines of the local Ethics Committee of National Research Lobachevsky State University of Nizhny Novgorod and guidelines of the local Ethics Committee of Ghent University (ECD19/35). The study was carried out in compliance with the ARRIVE guidelines**.**

Female C57BL/6 J mice (7–8 weeks old) and BALB/c Nude mice (Nude mice, 8–10 weeks old) were obtained from Charles River (CAnN.Cg-Foxn1^nu^/Crl, immunodeficient inbred) and were housed in specific pathogen-free conditions. Pz I-PDT and pz III-PDT were used as described above to induce cell death in fibrosarcoma MCA205 cells in vitro. Following cell death induction, the cells were cultivated for 20 − 24 h in complete culture medium, collected, washed once in PBS, and resuspended at the desired cell density in PBS. Mice were injected subcutaneously in the left flank with 5 × 10^5^ dying MCA205 cells, MCA205 cells subjected to F/T cycles, or with PBS. As a control, the mice were challenged on the opposite flank with 1 × 10^5^ live MCA205 cells on day 8. The challenge site was inspected for tumor appearance, and tumor growth was measured with a caliper for up to 4 weeks. If the tumors became necrotic or exceeded 2 cm^3^, the mice were sacrificed.

### Statistical analysis

GraphPad Prism (v.6.0) was used for statistical analysis. Cell death was analyzed by ANOVA, followed by t-criteria with Bonferroni correction. In the experiments in which DAMPs analysis and phagocytosis assay were done, statistical analysis of the results of cell death was done with the Kolmogorov–Smirnov test. Kaplan–Meier survival curves showing the timeline of tumor development were analyzed by log-rank Mantel–Cox test. In the coculture experiments where maturation and activation of BMDCs were analyzed the statistical significance was calculated by a multiple t-test using Sidak-Bonferroni method. Differences between tumor volumes in the in vivo prophylactic tumor vaccination experiments were analyzed by a non-parametric Mann–Whitney test.

## Supplementary Information

Supplementary Information.
