# Supplementary material for: Novel porphyrazine-based photodynamic anti-cancer therapy induces immunogenic cell death
Source: Sci Rep. 2021 Mar 30;11:7205. doi: 10.1038/s41598-021-86354-4 (PMC8010109; doi:10.1038/s41598-021-86354-4)
Supplement: Supplementary file 1 — Supplementary Information. [file 41598_2021_86354_MOESM1_ESM.pptx]

## Slide 1
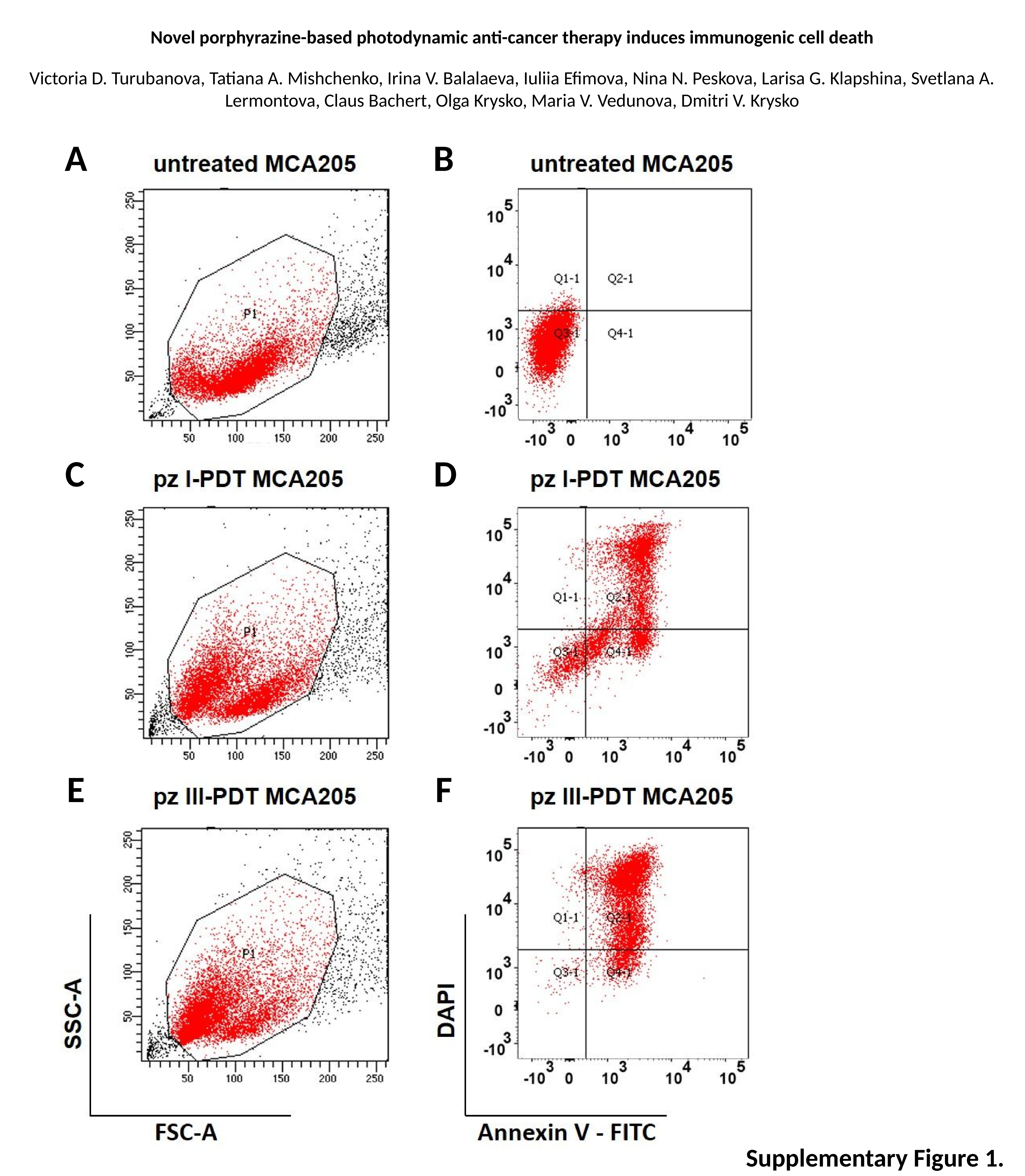

Novel porphyrazine-based photodynamic anti-cancer therapy induces immunogenic cell death
Victoria D. Turubanova, Tatiana A. Mishchenko, Irina V. Balalaeva, Iuliia Efimova, Nina N. Peskova, Larisa G. Klapshina, Svetlana A. Lermontova, Claus Bachert, Olga Krysko, Maria V. Vedunova, Dmitri V. Krysko
A
B
C
D
E
F
Supplementary Figure 1.

## Slide 2
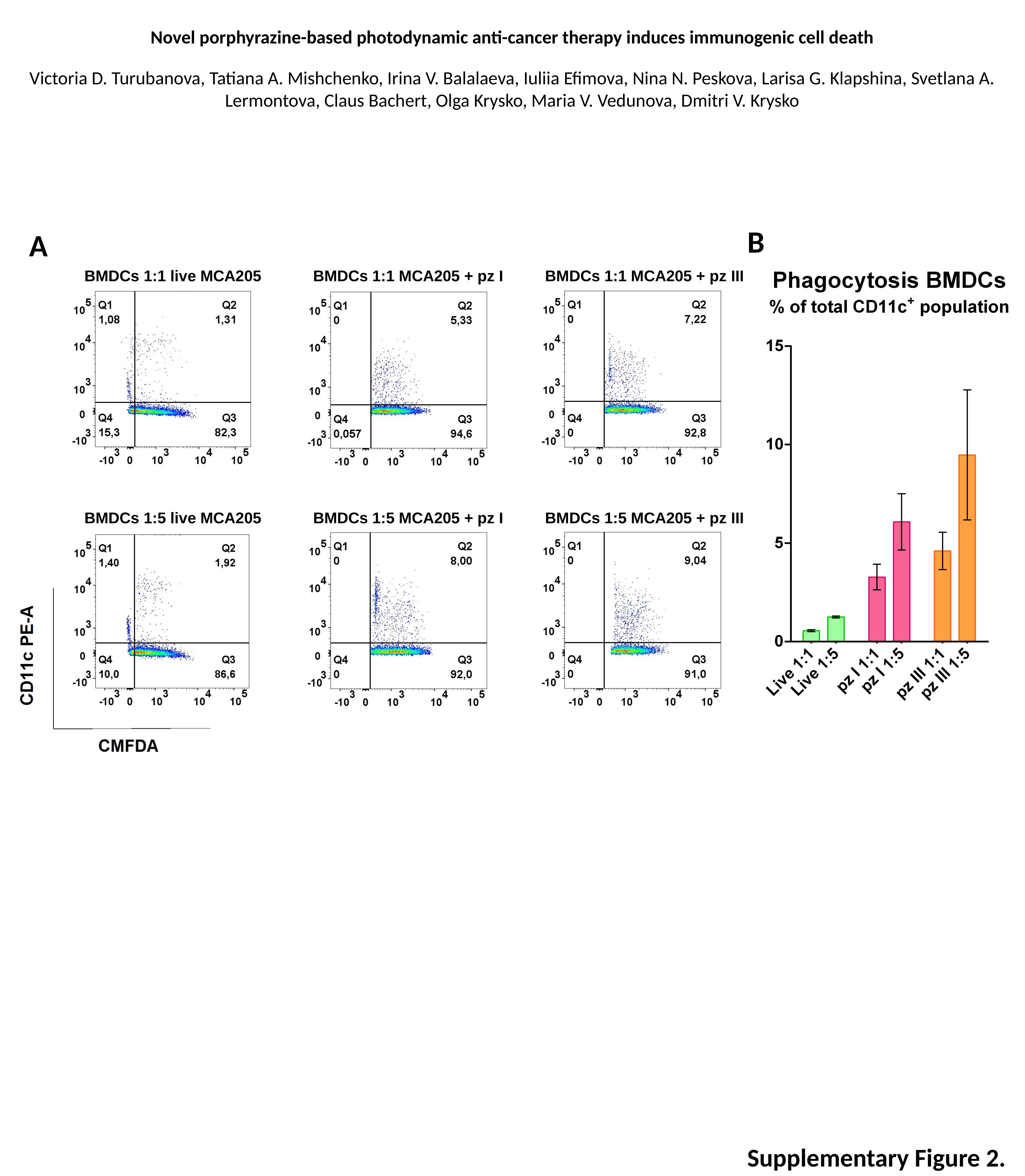

Novel porphyrazine-based photodynamic anti-cancer therapy induces immunogenic cell death
Victoria D. Turubanova, Tatiana A. Mishchenko, Irina V. Balalaeva, Iuliia Efimova, Nina N. Peskova, Larisa G. Klapshina, Svetlana A. Lermontova, Claus Bachert, Olga Krysko, Maria V. Vedunova, Dmitri V. Krysko
B
A
BMDCs 1:1 live MCA205
BMDCs 1:1 MCA205 + pz I
BMDCs 1:1 MCA205 + pz III
BMDCs 1:5 live MCA205
BMDCs 1:5 MCA205 + pz I
BMDCs 1:5 MCA205 + pz III
Supplementary Figure 2.

## Slide 3
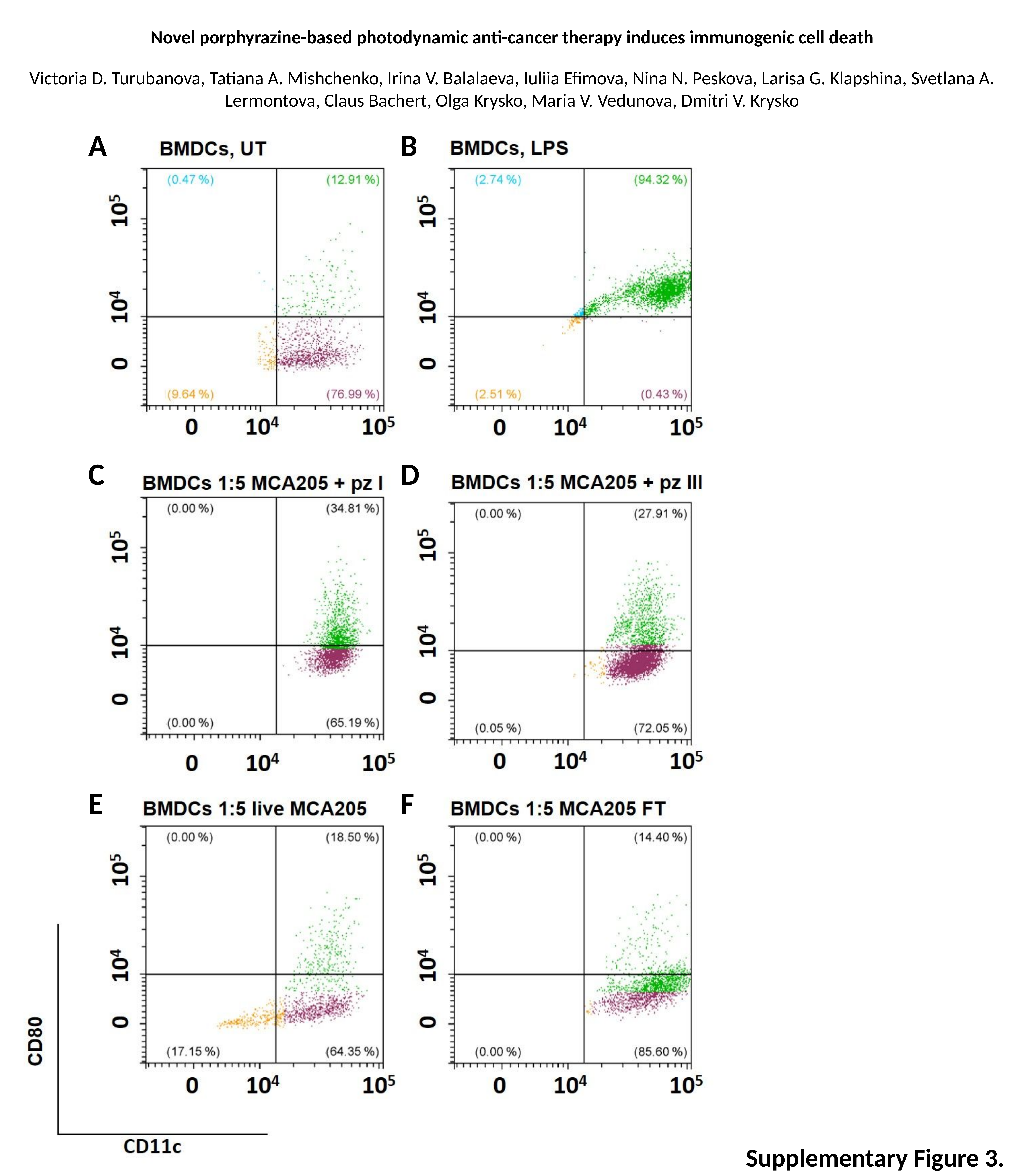

Novel porphyrazine-based photodynamic anti-cancer therapy induces immunogenic cell death
Victoria D. Turubanova, Tatiana A. Mishchenko, Irina V. Balalaeva, Iuliia Efimova, Nina N. Peskova, Larisa G. Klapshina, Svetlana A. Lermontova, Claus Bachert, Olga Krysko, Maria V. Vedunova, Dmitri V. Krysko
A
B
D
C
E
F
Supplementary Figure 3.

## Slide 4
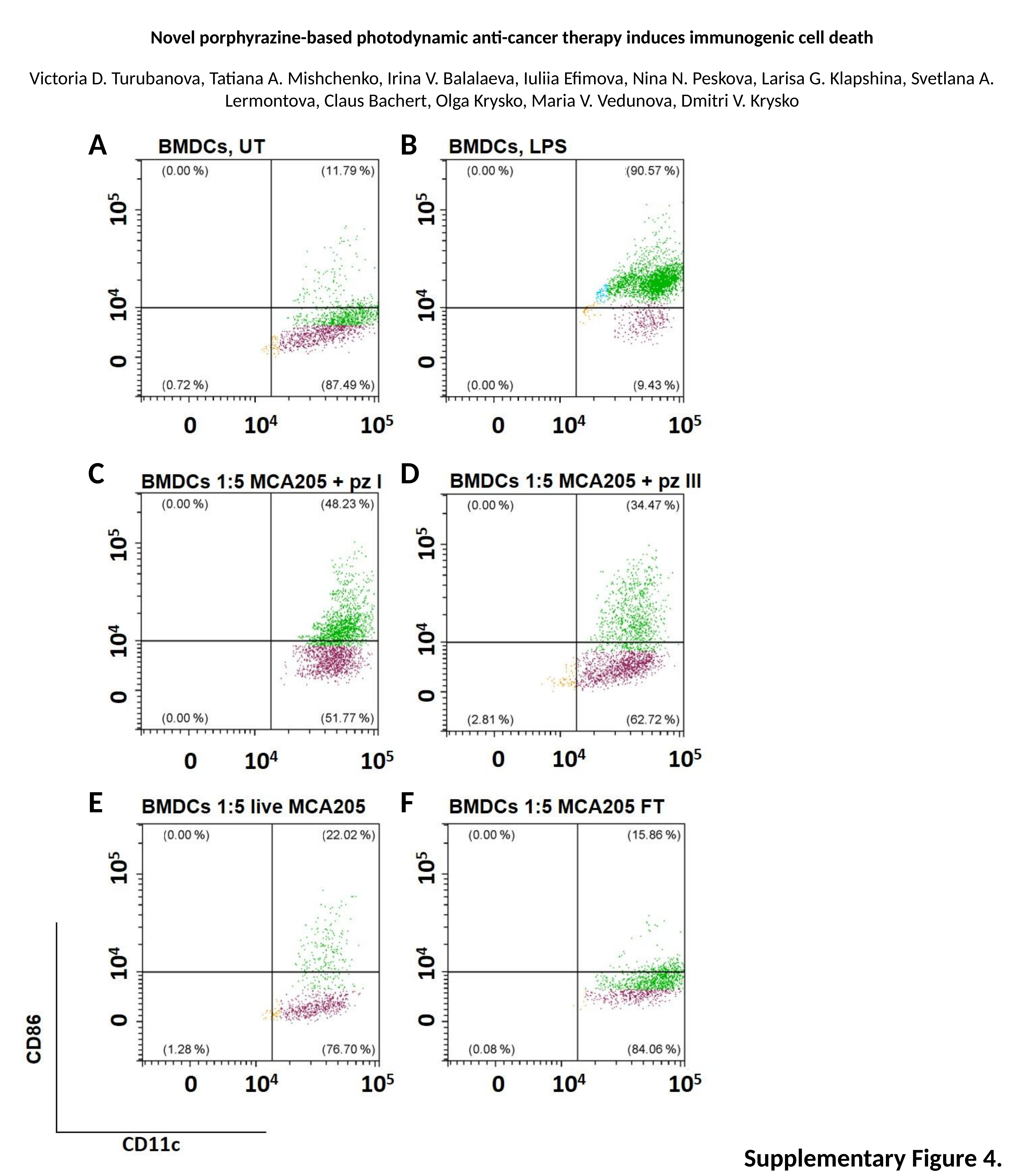

Novel porphyrazine-based photodynamic anti-cancer therapy induces immunogenic cell death
Victoria D. Turubanova, Tatiana A. Mishchenko, Irina V. Balalaeva, Iuliia Efimova, Nina N. Peskova, Larisa G. Klapshina, Svetlana A. Lermontova, Claus Bachert, Olga Krysko, Maria V. Vedunova, Dmitri V. Krysko
A
B
D
C
E
F
Supplementary Figure 4.

## Slide 5
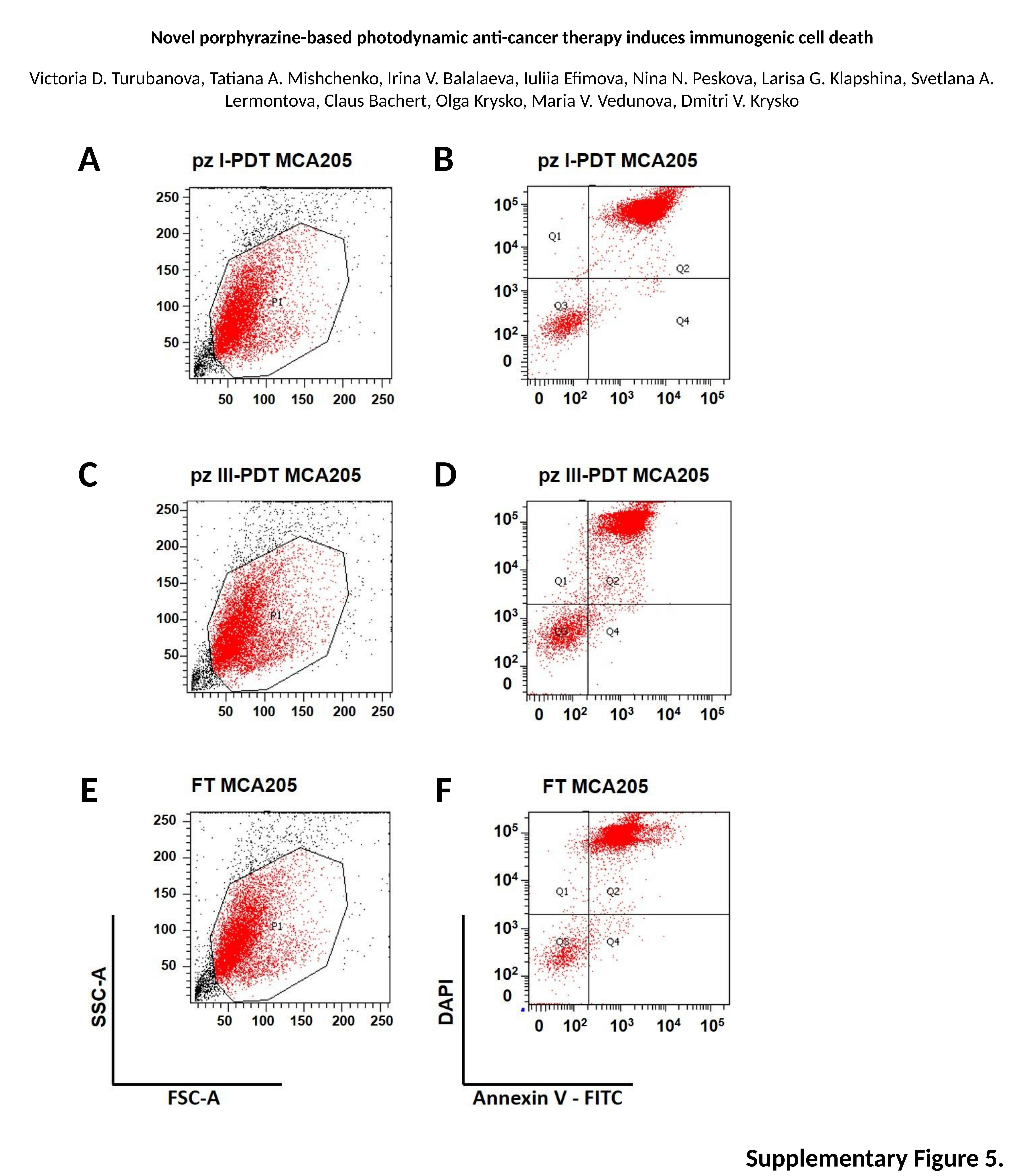

Novel porphyrazine-based photodynamic anti-cancer therapy induces immunogenic cell death
Victoria D. Turubanova, Tatiana A. Mishchenko, Irina V. Balalaeva, Iuliia Efimova, Nina N. Peskova, Larisa G. Klapshina, Svetlana A. Lermontova, Claus Bachert, Olga Krysko, Maria V. Vedunova, Dmitri V. Krysko
A
B
C
D
E
F
Supplementary Figure 5.
